# Neuroplasticity Meets Artificial Intelligence: A Hippocampus-Inspired Approach to the Stability–Plasticity Dilemma

**DOI:** 10.3390/brainsci14111111

**Published:** 2024-10-31

**Authors:** Thorsten Rudroff, Oona Rainio, Riku Klén

**Affiliations:** Turku PET Centre, University of Turku and Turku University Hospital, 20520 Turku, Finland; ormrai@utu.fi (O.R.); riku.klen@utu.fi (R.K.)

**Keywords:** artificial intelligence, hippocampus, neocortex, stability–plasticity dilemma, continual learning, memory consolidation, brain-inspired computing

## Abstract

The stability–plasticity dilemma remains a critical challenge in developing artificial intelligence (AI) systems capable of continuous learning. This perspective paper presents a novel approach by drawing inspiration from the mammalian hippocampus–cortex system. We elucidate how this biological system’s ability to balance rapid learning with long-term memory retention can inspire novel AI architectures. Our analysis focuses on key mechanisms, including complementary learning systems and memory consolidation, with emphasis on recent discoveries about sharp-wave ripples and barrages of action potentials. We propose innovative AI designs incorporating dual learning rates, offline consolidation, and dynamic plasticity modulation. This interdisciplinary approach offers a framework for more adaptive AI systems while providing insights into biological learning. We present testable predictions and discuss potential implementations and implications of these biologically inspired principles. By bridging neuroscience and AI, our perspective aims to catalyze advancements in both fields, potentially revolutionizing AI capabilities while deepening our understanding of neural processes.

## 1. Introduction

The quest to create artificial intelligence (AI) systems capable of continuous learning while maintaining previously acquired knowledge remains one of the grand challenges in machine learning. This challenge, known as the stability–plasticity dilemma, lies at the heart of developing AI systems that can adapt to new information without catastrophically forgetting past learning [1].

**Figure 1:**
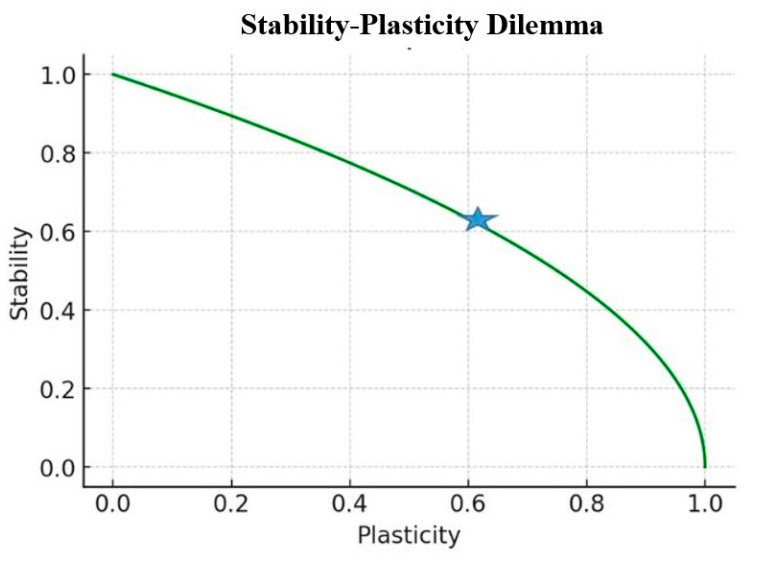
The Stability-plasticity trade-off in learning systems. The graph illustrates the fundamental trade-off between stability (y-axis) and plasticity (x-axis) in learning systems. The green curve represents the Pareto frontier, showing the optimal trade-off where increasing one capability necessarily reduces the other. The blue dot marks the inflection point, where the trade-off dynamics shift from gradual to more pronounced changes, representing a critical balance between stability and plasticity.

Figure 1 illustrates the fundamental trade-off between stability (the ability to retain existing knowledge) and plasticity (the capacity to acquire new information) in learning systems. The x-axis represents increasing plasticity from left to right, while the y-axis represents increasing stability from bottom to top. This Pareto Frontier Graph shows the optimal trade-off between stability and plasticity. The curve indicates the efficient frontier where improvements in one dimension lead to compromises in the other.

On the Pareto frontier, the inflection point represents the location where there is a change in the curve’s curvature. In the context of Pareto optimality, the inflection point signifies the transition between two regimes of trade-offs between objectives: before this point, it is possible to achieve relatively significant gains in one objective with only a small loss in the other, whereas after it, making further improvements becomes increasingly costly, and the trade-offs become more pronounced. The inflection point is marked with an asterisk.

Recent advances in AI, such as DeepMind’s muZero Schrittwieser and colleagues [2], have demonstrated impressive capabilities in learning and decision-making across various domains. However, these systems still struggle with balancing the need for plasticity to acquire new knowledge and stability to retain existing information.

In the realm of neuroscience, the mammalian brain, particularly the hippocampus–cortex system, provides a compelling model for addressing this dilemma. This biological system has evolved to effectively balance the rapid learning of new experiences with the gradual integration of knowledge into long-term memory [3]. Recent work by Wirtshafter and Wilson [4] has highlighted intriguing parallels between the functions of advanced AI systems like muZero and hippocampal processing, suggesting a bidirectional opportunity for insight between AI and neuroscience.

Building on these recent developments, this perspective paper argues that a deeper understanding and implementation of hippocampus–cortex-inspired architectures could lead to significant advancements in AI systems’ ability to manage the stability–plasticity trade-off. It is proposed that by mimicking the dual fast and slow learning systems observed in the hippocampus and neocortex, future AI architectures could achieve more effective continual learning while maintaining the ability to generalize across contexts.

This perspective paper will explore the following:

1. The current state of the stability–plasticity dilemma in AI and its parallels in neuroscience.
2. Key features of the hippocampus–cortex system relevant to addressing this dilemma, including complementary learning systems [5], memory replay [6], and contextual representation [7].
3. Proposed AI architectures inspired by the hippocampus–cortex system, emphasizing dual learning rates and offline consolidation mechanisms.
4. Potential implications and challenges in implementing these biologically inspired approaches in AI systems.
5. Future directions for research at the intersection of neuroscience and AI, with a focus on continual learning and knowledge integration.

By examining how the brain solves the stability–plasticity dilemma, the aim of this perspective is to inspire novel approaches in AI that could lead to more adaptive and generalizable learning systems. This interdisciplinary perspective not only offers potential advancements in AI but also provides a framework for testing and refining our understanding of hippocampal and cortical functions in biological learning and memory.

This perspective bridges neuroscience and artificial intelligence, offering novel insights for advancing machine learning through an understanding of biological neural systems. Given its interdisciplinary nature, we will introduce and explain key terminology from both neuroscience and computer science as it appears in the text. This approach aims to make the content accessible to readers from diverse backgrounds, fostering a shared understanding of complex concepts such as non-REM sleep, sharp-wave ripples (SWRs), and barrages of action potentials (BARRs) alongside their potential analogues in artificial neural networks. By explicitly connecting these biological mechanisms to computational concepts, we hope to facilitate the cross-pollination of ideas between these two rapidly advancing fields.

## 2. The Hippocampus–Cortex System

The hippocampus–cortex system is a crucial neural architecture that plays a central role in learning, memory formation, and spatial navigation. This system exemplifies a solution to the stability–plasticity dilemma through its complementary fast and slow learning mechanisms [8]. Understanding the intricacies of this biological system can provide valuable insights for developing more adaptive AI architectures.

**Figure 2:**
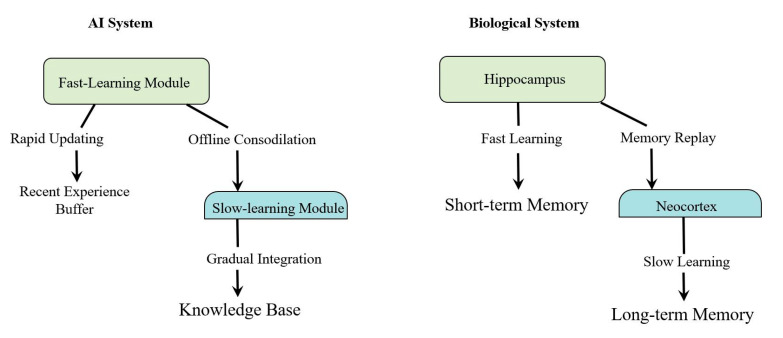
Parallel architecture of artificial and biological learning systems. Comparison between AI and biological learning systems, illustrating their analogous dual-stream processing. Both systems feature fast-learning components (Fast-Learning Module/Hippocampus) that interact with slow-learning components (Slow-learning Module/Neocortex) through memory consolidation. The biological system employs bidirectional crosstalk between the hippocampus and neocortex during memory replay, enabling systematic integration of new information while maintaining existing knowledge.

Figure 2 illustrates a striking parallel between biological and artificial learning systems, highlighting how both employ a two-stream approach to memory formation and learning. At its core, both systems rely on a crucial interplay between fast and slow learning mechanisms, centered on memory consolidation.

In biological systems, the hippocampus serves as the initial, fast-learning center that rapidly captures new experiences. Through a process called memory consolidation, these experiences are gradually transferred to the neocortex, which acts as a slow-learning module responsible for long-term memory storage. Importantly, this consolidation process involves bidirectional crosstalk between the hippocampus and neocortex rather than a simple one-way flow of information. Through memory replay, this back-and-forth communication allows short-term memories to be systematically integrated into lasting knowledge while also allowing existing knowledge to influence how new information is processed and stored.

Mirroring this biological architecture, artificial systems implement a similar dual-stream approach. A fast-learning module quickly processes new information, temporarily storing recent experiences in a buffer. Through offline consolidation, this information is gradually integrated into a slow-learning module. This careful process ultimately leads to the formation of a stable knowledge base, much like how human long-term memories are formed.

The key to both systems lies in the delicate balance between rapid acquisition and gradual consolidation. Memory consolidation–whether between the hippocampus and neocortex in biological systems or between fast and slow-learning modules in AI systems–ensures that new information is meaningfully integrated without disrupting existing knowledge. This parallel organization reveals how artificial systems have been designed to emulate the time-tested architecture of biological learning.

### 2.1. Key Concepts and Terminology

To facilitate a comprehensive understanding of the hippocampus–cortex system and its relevance to AI, it is crucial to define and explain several key concepts and terms. These concepts, drawn from neuroscience, form the foundation of our discussion on brain-inspired AI approaches. By providing clear explanations of these terms, we aim to bridge the gap between neuroscience and computer science, enabling readers from both fields to engage fully with the interdisciplinary nature of this work. The following definitions and explanations will be referenced throughout the subsequent discussions on memory consolidation, learning, and AI design.

Non-REM vs REM sleep: Non-REM (Non-Rapid Eye Movement) sleep is a phase of sleep characterized by slower brain waves and reduced neural activity. It is divided into three stages, with the deepest stage (slow-wave sleep) being crucial for memory consolidation. In contrast, REM (Rapid Eye Movement) sleep is characterized by rapid eye movements and increased brain activity similar to that of wakefulness and is associated with vivid dreaming [9,10].

SWRs: SWRs are brief, high-frequency oscillations observed in the hippocampus, primarily during non-REM sleep and quiet wakefulness. They are associated with the reactivation and consolidation of recently acquired memories [11,12]. In the context of neural networks, SWRs can be thought of as burst-like activations that strengthen specific connection patterns [4].

BARRs: BARRs are recently discovered synchronized bursts of neural activity, primarily involving CA2 pyramidal cells and certain interneurons in the hippocampus. They occur during non-REM sleep and appear to play a complementary role to SWRs in memory processing [13]. In artificial neural network terms, BARRs could be analogous to coordinate inhibitory signals that help maintain network stability and prevent the over-strengthening of recently potentiated connections [4].

### 2.2. Complementary Learning Systems

The hippocampus and neocortex work in tandem as complementary learning systems (CLS) [14]. The hippocampus, with its ability to rapidly form detailed memories of specific experiences, serves as a fast-learning system. In contrast, the neocortex gradually integrates information over time, extracting general patterns and rules from repeated experiences, functioning as a slow-learning system [15].

This dual-system approach allows for the quick acquisition of new information without disrupting existing knowledge, addressing a key aspect of the stability–plasticity dilemma. The hippocampus can quickly encode new experiences, while the neocortex slowly incorporates this information into its existing knowledge structures [16].

### 2.3. Biological Solutions to the Stability–Plasticity Dilemma

The stability–plasticity dilemma is a fundamental challenge in both biological and artificial learning systems. The brain’s solution to this dilemma, particularly in the hippocampus–cortex system, offers valuable insights for AI design. Recent research by Karaba and colleagues [13] has shed new light on the intricate mechanisms the brain employs to balance the need for plasticity (learning new information) with stability (retaining existing knowledge).

#### 2.3.1. Complementary Network Events: SWRs and BARRs

The hippocampus utilizes two complementary types of network events during non-REM sleep to address the stability–plasticity dilemma: SWRs and the newly discovered BARRs.

1. SWRs: SWRs have long been recognized as crucial for memory consolidation. During these events, neurons that were active during recent experiences are reactivated in a time-compressed manner [13]. This reactivation strengthens the neural connections associated with new memories, facilitating their integration into existing knowledge structures in the neocortex.
2. BARRs: Karaba and colleagues [13] discovered that BARRs play a complementary role to SWRs. BARRs involve the synchronized firing of a subset of CA2 pyramidal cells and cholecystokinin-expressing (CCK+) basket cells. Crucially, neurons and assemblies that were active during learning and subsequently reactivated during SWRs are inhibited during BARRs.

#### 2.3.2. Balancing Act: Selective Strengthening and Network Stability

The alternating pattern of activation (during SWRs) and inhibition (during BARRs) provides a sophisticated mechanism for addressing the stability–plasticity dilemma:

1. Plasticity through SWRs: SWRs promote plasticity by selectively reactivating and strengthening neural patterns associated with recent, important experiences. This process allows for the rapid acquisition and initial consolidation of new memories.
2. Stability through BARRs: BARRs contribute to network stability by inhibiting the same neurons and assemblies that were previously activated. This inhibition may serve several crucial functions: (a) Preventing runaway excitation: By dampening the activity of recently strengthened neural patterns, BARRs may prevent the excessive amplification of new memories, which could otherwise lead to network instability. (b) Maintaining relative synaptic weights: The inhibition during BARRs might help preserve the relative importance of different memories by preventing the over-strengthening of the most recent experiences. (c) Facilitating integration: By temporarily suppressing recently acquired information, BARRs may create opportunities for older, related memories to be activated and integrated with new learning.
3. Dynamic regulation: Karaba and colleagues [13] found that the initial increase in reactivation during SWRs gradually returned to baseline levels through sleep. This trend was abolished when CCK+ basket cells were silenced during BARRs, resulting in higher synchrony of the CA1 assemblies and impaired memory consolidation. This suggests that the interplay between SWRs and BARRs is crucial for appropriately balancing synaptic strengthening and network stability over time.

#### 2.3.3. Implications for Artificial Intelligence

The hippocampus–cortex system, particularly the recent discovery of BARRs [13], offers several key principles that could inform AI design and potentially address the stability–plasticity dilemma in artificial systems:

1. Dual Learning Rates and Complementary Processes: The combination of fast hippocampal learning with slow cortical integration provides a model for balancing quick adaptation with long-term stability. AI systems could implement dual-memory architectures with different learning rates to mimic this biological strategy [17]. Furthermore, inspired by the complementary roles of SWRs and BARRs, AI systems could incorporate alternating phases of activation and targeted inhibition during the consolidation process.
  Implementation: Design a neural network with two interacting components: a fast-learning module (analogous to the hippocampus) and a slow-learning module (analogous to the neocortex). The training regime should alternate between strengthening recent patterns (inspired by SWRs) and selectively inhibiting these same patterns (inspired by BARRs) to prevent overfitting and maintain network stability.
2. Offline Consolidation with Selective Inhibition: The use of replay for gradual knowledge integration during periods of rest or low activity could inspire similar mechanisms in AI [18,19]. This could involve periodic “sleep” phases where the AI system consolidates recent experiences into a more stable knowledge base. The discovery of BARRs suggests that this process should include not only the reactivation of recent experiences but also their selective inhibition.
  Implementation: Design an AI system with dedicated offline processing periods that implement both prioritized experience replay (inspired by SWRs) and a novel “selective inhibition” mechanism (inspired by BARRs) that temporarily downregulates the influence of recently strengthened connections.
3. Dynamic Regulation of Plasticity: The temporal dynamics observed in the interplay between SWRs and BARRs, where reactivation strength gradually returns to the baseline, suggesting that AI systems could benefit from the dynamic regulation of plasticity over time.
  Implementation: Develop a time-dependent learning rate that starts high for new information and gradually decreases, mimicking the biological return to the baseline activation. This could be coupled with a complementary inhibition strength that increases over time for recently learned patterns.
4. Circuit-Specific Mechanisms: The distinct roles of different neural subtypes (e.g., CA2 pyramidal cells and CCK+ basket cells) in BARRs highlight the potential benefits of incorporating diverse, functionally specialized components in AI architectures.
  Implementation: Design AI architectures with functionally distinct subnetworks that play different roles in learning and consolidation. For example, one subnetwork could specialize in the rapid encoding of new information, while another focuses on selectively inhibiting recent patterns to maintain overall network stability.
5. Contextual Representation with Balanced Plasticity: The hippocampus’s ability to form distinct representations of similar experiences (pattern separation) while also generalizing across contexts (pattern completion) could inform the development of more flexible AI learning algorithms [20,21]. The addition of BARR-inspired mechanisms could help maintain the stability of these representations over time.
  Implementation: Develop AI systems with enhanced abilities to create distinct contextual representations, coupled with a mechanism for selectively strengthening or inhibiting these representations based on their recency and importance.
6. Hierarchical Organization with Regulated Plasticity: The progression from specific episodic memories in the hippocampus to more abstract, semantic knowledge in the cortex suggests a hierarchical organization of knowledge. This principle could be applied to develop AI architectures that build increasingly abstract representations over time, with BARR-inspired mechanisms helping to regulate plasticity at different levels of the hierarchy.
  Implementation: Create a hierarchical neural network where lower levels capture specific details, and higher levels represent more general concepts. Implement different plasticity rules at each level, with BARR-inspired inhibition more strongly regulating lower, more plastic levels to prevent overfitting to recent experiences.

By incorporating these biologically inspired principles, including the newly discovered BARR mechanism, AI systems might better navigate the stability–plasticity dilemma, achieving both rapid learning and long-term knowledge retention without catastrophic forgetting. Moreover, these approaches could lead to AI systems with more human-like learning capabilities, including improved transfer learning, few-shot learning, and continual learning [8,14].

The integration of BARR-like mechanisms represents a novel approach to addressing the stability–plasticity dilemma. By mimicking the brain’s sophisticated balance between strengthening new memories and preventing network oversaturation, we may develop AI systems that are both more adaptive and more stable, capable of continuous learning in complex, changing environments.

**Figure 3:**
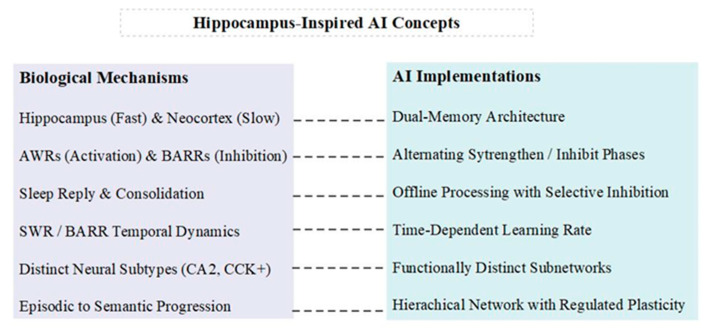
Hippocampus-inspired AI concepts. This diagram compares key features of the hippocampus–cortex system (**left**) with potential AI implementations (**right**). It illustrates how biological mechanisms like dual learning rates, memory consolidation, and hierarchical organization inspire corresponding AI designs, highlighting the potential for neuroscience-informed advancements in artificial intelligence.

To visually summarize the key concepts discussed in this section, Figure 3 illustrates the parallels between biological mechanisms in the hippocampus–cortex system and their potential AI implementations.

This diagram presents a side-by-side comparison of biological mechanisms and their corresponding AI implementations. The dual learning rates of the hippocampus and neocortex inspire dual-memory architectures in AI systems. The interplay between SWRs and BARRs suggests alternating strengthening and inhibiting phases for AI training regimes. The process of offline consolidation during sleep informs offline processing strategies with selective inhibition in AI.

The temporal dynamics of SWRs and BARRs inspire the implementation of time-dependent learning rates in artificial systems. The specialized circuits involving distinct neural subtypes suggest the potential benefits of functionally distinct subnetworks in AI architectures. Finally, the hierarchical organization of memory inspires hierarchical networks with regulated plasticity in AI designs.

This visual representation underscores the rich potential for cross-pollination between neuroscience and artificial intelligence. By drawing inspiration from the sophisticated mechanisms of the brain, it becomes possible to develop more adaptive, robust, and efficient AI systems that better navigate the challenges of continuous learning and knowledge integration.

#### 2.3.4. Neuronal Representations and Plasticity

At the cellular level, the hippocampus exhibits remarkable plasticity and complex neuronal dynamics that contribute to its role in learning and memory. Recent research has revealed even more intricate mechanisms that fine-tune this plasticity, balancing the need for rapid learning with network stability.

Place cells in the hippocampus which fire when an animal is in specific locations and can rapidly form new representations of novel environments [22]. This quick adaptation allows for the immediate encoding of new spatial contexts [23]. The entorhinal cortex, which serves as the main interface between the hippocampus and neocortex, contains grid cells that provide a coordinate system for spatial navigation [24]. These cells maintain more stable representations across environments, potentially serving as a universal spatial map onto which specific experiences can be anchored [25].

Karaba and colleagues [13] have revealed additional layers of complexity in hippocampal neuronal dynamics, particularly in the interplay between different cell types during memory consolidation. They identified a subset of CA2 pyramidal cells that exhibit distinctive firing patterns during non-REM sleep, contributing to BARRs. These CA2 neurons, along with cholecystokinin-expressing (CCK+) basket cells in CA1, play a crucial role in regulating plasticity:

1. Cell-type specific roles: The study found that deep CA2 pyramidal cells were more active during BARRs, while superficial CA2 cells were more active during SWRs. This anatomical and functional segregation suggests highly specialized roles for different neuronal subpopulations in memory processing.
2. Differential plasticity regulation: During BARRs, CA1 neurons that had increased their activity during learning were selectively inhibited. This mechanism appears to provide a counterbalance to the strengthening of synapses that occurs during SWR-associated replay.
3. Dynamic plasticity modulation: The alternation between SWRs and BARRs creates a dynamic modulation of neuronal plasticity. Synapses associated with recent learning are strengthened during SWRs and then selectively suppressed during BARRs, potentially preventing runaway excitation and maintaining network stability.
4. Time-dependent plasticity changes: Karaba and colleagues [13] observed that the reactivation of learning-related neural patterns during SWRs gradually decreased over the course of sleep, returning to baseline levels. This suggests a time-dependent regulation of plasticity, where initial strong reactivations give way to more stabilized representations.
5. Interneuron-mediated plasticity control: The study highlighted the crucial role of CCK+ interneurons in regulating plasticity. These cells were highly active during BARRs but not during SWRs, suggesting they play a key role in the selective inhibition of recently potentiated synapses.

This intricate interplay between different cell types and network events (SWRs and BARRs) provides a biological solution to the stability–plasticity dilemma. It allows for the rapid formation of new memories through the plasticity of place cells and the reactivation during SWRs while also incorporating mechanisms to stabilize these memories and prevent overexcitation through the selective inhibition during BARRs.

Understanding these complex dynamics of neuronal representations and plasticity in the hippocampus not only deepens our knowledge of biological memory systems but also offers inspiration for developing more sophisticated AI architectures. Future AI systems might benefit from incorporating analogous mechanisms of dynamic, cell-type-specific, and temporally regulated plasticity to achieve a better balance between rapid learning and long-term stability.

### 2.4. Synaptic Plasticity and Long-Term Potentiation

Long-Term Potentiation (LTP) is a fundamental mechanism of synaptic plasticity that plays a crucial role in learning and memory formation, particularly in the hippocampus. First discovered by Terje Lømo in 1966 and later characterized by Bliss and Lømo in 1973 [26], LTP refers to a long-lasting enhancement in signal transmission between two neurons following the high-frequency stimulation of a chemical synapse [22].

LTP is widely considered to be one of the major cellular mechanisms underlying learning and memory [23]. It occurs primarily through the activation of N-methyl-D-aspartate (NMDA) receptors, leading to an influx of calcium ions and the subsequent strengthening of synaptic connections [24]. This process involves both pre- and post-synaptic changes, including increased neurotransmitter release and upregulation of AMPA receptors [25].

In the context of AI and machine learning, LTP-inspired mechanisms could potentially enhance the learning capabilities of artificial neural networks, particularly in the areas of rapid learning and memory consolidation [27]. By incorporating principles of LTP, AI systems might better emulate the brain’s ability to form and strengthen connections based on repeated activation, potentially addressing aspects of the stability–plasticity dilemma [28].

Future research integrating LTP-like mechanisms into AI architectures could lead to more efficient and adaptive learning algorithms, closer to the brain’s remarkable capacity for continuous learning and memory formation [29].

### 2.5. Memory Consolidation and Replay

A key mechanism in the hippocampus–cortex system is memory consolidation, where information initially encoded in the hippocampus is gradually transferred to the neocortex for long-term storage. This process often occurs during sleep or periods of rest through a phenomenon known as replay [30].

During replay, the hippocampus “replays” recently encoded experiences, often in a time-compressed manner. This replay is coordinated with neocortical activity, facilitating the gradual incorporation of new information into existing cortical networks [6]. This process allows for the integration of new memories without catastrophic interference with existing knowledge [16].

**Figure 4:**
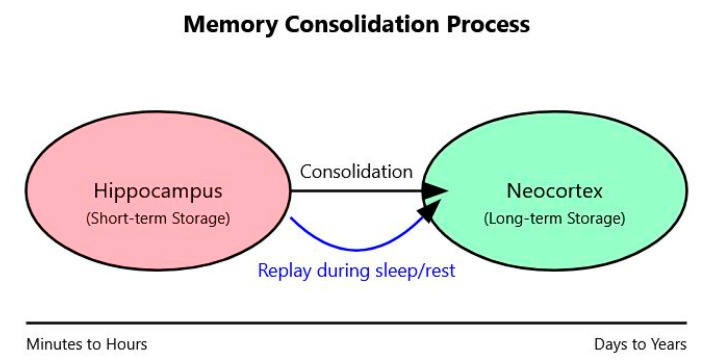
The process of memory consolidation, showing how information is transferred from short-term storage in the hippocampus to long-term storage in the neocortex, facilitated by replay during periods of rest and sleep.

Figure 4 illustrates the process of memory consolidation in the brain, specifically the transfer of information from short-term storage in the hippocampus to long-term storage in the neocortex. The left side of the diagram shows the hippocampus, represented by a pink ellipse and labeled as the site of short-term storage. On the right, a green ellipse represents the neocortex, labeled as the site of long-term storage.

A black arrow connecting these two regions represents the general process of memory consolidation, indicating the transfer of information from the hippocampus to the neocortex over time. Below this, a blue curved arrow represents the process of memory replay that occurs during sleep and rest periods. This replay is a crucial mechanism in the consolidation process, allowing for the reactivation and strengthening of neural patterns associated with recent experiences.

The timeline at the bottom of the diagram provides a sense of the temporal scale of this process. It shows that initial encoding in the hippocampus occurs within minutes to hours, while the full consolidation of memories in the neocortex can take days to years.

This visualization encapsulates the dynamic nature of memory formation and storage in the brain, highlighting the complementary roles of the hippocampus and neocortex in this process.

As discussed earlier, SWRs and BARRs play complementary roles in memory consolidation. During this process, the reactivation of neural patterns during SWRs is balanced by the selective inhibition provided by BARRs, allowing for the integration of new information without disrupting existing neural networks. This delicate balance enables the brain to maintain network stability while still remaining plastic enough to encode new experiences. The alternating pattern of activation and inhibition helps prevent the overexcitation of recently strengthened synapses, potentially preserving the relative importance of different memories and facilitating their gradual incorporation into long-term storage. This mechanism exemplifies the brain’s sophisticated solution to the stability–plasticity dilemma, providing a model for AI systems to achieve continuous learning without catastrophic forgetting.

In AI, the concepts of “rest” and “sleep” manifest as specific processing phases inspired by biological memory consolidation:

1. Offline Processing: AI systems engage in internal computations to consolidate and optimize learning, analogous to memory consolidation during sleep [9].
2. Experience Replay: Inspired by hippocampal replay, AI systems reprocess previous experiences to reinforce learning and integrate new information with existing knowledge [31].
3. Model Consolidation: In continual learning setups, “sleep” phases consolidate knowledge from recent learning into a more stable, general model, mirroring hippocampal–neocortical dialogue [15].
4. Regularization and Pruning: AI systems maintain network efficiency through processes analogous to synaptic homeostasis in neuroscience [31].

These processes, while inspired by biological rest and sleep, are computational analogues serving similar functional roles: consolidating learning, integrating new information, and optimizing system performance.

Crucially, these “rest and sleep” phases in AI can implement mechanisms inspired by the interplay between SWRs and BARRs observed in hippocampal memory consolidation. AI architectures could incorporate alternating phases of strengthening recent memories (inspired by SWRs) and selective inhibition (inspired by BARRs) during offline processing. This approach aims to balance rapid learning with long-term stability, addressing key challenges in continual learning and knowledge integration without catastrophic forgetting.

### 2.6. Contextual Representation and Pattern Separation

The hippocampus is adept at forming distinct representations of similar experiences, a process known as pattern separation [20]. This ability allows for the differentiation of contexts and the formation of unique memory traces, even when experiences share many common features.

Conversely, the hippocampus can also perform pattern completion, where partial cues can trigger the recall of entire memory representations [21]. This balance between pattern separation and completion allows for both specific memory recall and generalization across similar experiences.

## 3. Current Challenges in AI: Catastrophic Forgetting

As AI systems advance, they face challenges that mirror those in biological learning systems, particularly with continuous learning in complex environments. A critical challenge is catastrophic forgetting, which directly relates to the stability–plasticity dilemma central to this paper.

Catastrophic forgetting occurs when artificial neural networks rapidly overwrite previously acquired knowledge when exposed to new data or tasks [32,33]. This challenge is particularly acute in scenarios requiring sequential learning from non-stationary data distributions, a common real-world requirement. Unlike the human brain, which can continually acquire new knowledge without significantly disrupting existing memories, traditional artificial neural networks struggle to maintain previously learned information when adapting to new tasks [18].

Several approaches to mitigate catastrophic forgetting have been proposed, with some drawing inspiration from hippocampal function:

1. Elastic Weight Consolidation (EWC): This method slows down learning on weights crucial to previously seen tasks, akin to how the brain consolidates important memories [33].
2. Memory replay techniques: Inspired by hippocampal replay, these methods periodically revisit and retrain on past experiences, reinforcing previously learned knowledge [34].
3. Model storage and revisiting: This approach involves saving snapshots of the model at various training stages and selectively fine-tuning them later, aligning with the concept of memory rehearsal in cognitive psychology [34].

These approaches, particularly memory replay techniques, demonstrate how hippocampus-inspired mechanisms can address the stability–plasticity dilemma in AI. They aim to balance the preservation of existing knowledge (stability) with the ability to learn new information (plasticity).

However, a general solution to catastrophic forgetting across diverse AI applications remains an open challenge. The ideal solution would enable an AI system to continually learn and adapt without losing accumulated knowledge, mirroring the human brain’s ability to acquire new skills while retaining old ones. This goal aligns closely with the paper’s focus on leveraging hippocampal principles to create more adaptive and generalizable AI systems.

## 4. The Grand Challenge: Integrating Diverse AI Solutions

While we have explored various hippocampus-inspired solutions to AI challenges, a significant overarching challenge remains: effectively integrating these diverse approaches into a cohesive AI system. This integration challenge mirrors the complexity of the hippocampus–cortex system, where multiple specialized processes work in concert to produce adaptive learning and memory.

Key aspects of this integration challenge particularly relevant to hippocampus-inspired AI include the following:

1. Architectural Integration: Developing AI architectures that incorporate both fast and slow learning components, inspired by the hippocampus and neocortex respectively. This involves creating systems that can rapidly encode new experiences while gradually integrating this information into a stable knowledge base [5].
2. Balancing Plasticity and Stability: Implementing mechanisms that dynamically adjust the balance between rapid learning and long-term stability, analogous to the interplay between SWRs and BARRs observed in the hippocampus [13].
3. Context-Dependent Processing: Designing systems that can form distinct representations for similar experiences (pattern separation) while also generalizing across contexts (pattern completion), mirroring hippocampal function [17,21].
4. Memory Consolidation and Replay: Incorporating offline processing periods that allow for the gradual integration of new information into existing knowledge structures, inspired by hippocampal replay during sleep [35].
5. Hierarchical Knowledge Organization: Developing architectures that support the progression from specific episodic memories to more abstract, semantic knowledge, reflecting the hippocampus–neocortex dialogue [3].

Addressing these integration challenges will require interdisciplinary approaches, combining insights from neuroscience, cognitive science, and machine learning. Promising directions include the following:

- Dual-process learning algorithms that alternate between phases of rapid acquisition and selective inhibition.
- Hierarchical neural networks with regulated plasticity at different levels.
- Meta-learning approaches that continuously adapt learning strategies based on task demands and past experiences.

Successfully integrating these hippocampus-inspired mechanisms could lead to AI systems that are more flexible, robust, and capable of human-like learning and generalization. As we continue to unravel the complexities of the hippocampus–cortex system, we may find new insights into solving the grand challenge of creating truly adaptive and generalizable AI.

## 5. Lessons from Neuroscience: Synthesizing Hippocampus-Inspired AI Concepts

Throughout this paper, we have explored various aspects of the hippocampus–cortex system and their potential applications in artificial intelligence. This chapter serves as a comprehensive synthesis of these neuroscience concepts and their implications for AI design. By consolidating these ideas, we aim to provide a clear overview of how the sophisticated mechanisms of the hippocampus–cortex system can inspire novel approaches to address current challenges in machine learning.

As we have discussed, the hippocampus–cortex system offers a rich source of inspiration for AI, having evolved to effectively balance rapid learning with long-term knowledge retention. Here, we will summarize the key biological principles explored earlier and their potential AI implementations, emphasizing the interdisciplinary nature of this research.

Central to our discussion has been the concept of complementary learning systems, introduced by McClelland and colleagues [3] and elaborated by O’Reilly and Norman [36]. This dual-system approach, balancing rapid acquisition with the gradual integration of knowledge, could inspire AI architectures with separate fast and slow learning components. As we explored earlier, such an approach has been implemented in recent AI models, like the work of Sprechmann and colleagues [37] on memory-based parameter adaptation.

We have also examined the hippocampus’s ability to perform pattern separation and completion, as described by Yassa and Stark [20] and Rolls [21]. These mechanisms could inspire more robust and flexible memory systems in AI, potentially implemented through sparse coding techniques or attractor dynamics in recurrent neural networks [38,39].

The distinction between episodic and semantic memory, discussed in relation to the work of Tulving [39] and Renoult and colleagues [40], suggests the potential for AI systems that better balance specific and general knowledge. This could be realized through hierarchical memory systems, possibly leveraging techniques from hierarchical reinforcement learning [41].

Memory consolidation and replay, a key process we explored earlier [35,42], could inspire offline learning mechanisms in AI. This might involve implementing “sleep” phases in AI systems, with prioritized experience replay as proposed by Schaul and colleagues [17].

We have also considered how the hippocampus’s context-dependent representations [43,44] could inspire improved context-aware learning in AI, potentially through attention mechanisms similar to those in transformer networks [45].

The concepts of hierarchical predictive processing [46,47] and error-driven learning [48] in the brain could inform more efficient and adaptive AI learning algorithms. Similarly, neurogenesis and synaptic pruning [49,50] suggest the potential for dynamic network architectures in AI [51,52].

We have explored how hippocampal oscillatory patterns [11,53] might inspire new training regimes in AI, possibly involving alternating “modes” or the periodic synchronization of distributed systems [54].

Finally, we discussed Long-Term Potentiation (LTP) [3,22,26], a fundamental mechanism of synaptic plasticity, and its potential to enhance learning capabilities in artificial neural networks [5,27], particularly in addressing the stability–plasticity dilemma.

In conclusion, by synthesizing these diverse yet interconnected concepts from neuroscience, we can envision AI systems that are more flexible, adaptive, and capable of continuous learning. However, as noted throughout our discussion, the direct implementation of these biological principles may not always be optimal in artificial systems. The goal is to extract key computational principles and creatively adapt them to the strengths and limitations of AI.

This interdisciplinary approach not only promises to enhance AI capabilities but also offers a unique lens through which to further our understanding of biological intelligence. As our understanding of the brain continues to evolve, so will the potential for neuroscience-inspired AI, holding the promise of revolutionizing both fields in the years to come.

## 6. Future Directions: The Promise and Challenges of Brain-Inspired AI

The principles gleaned from the hippocampus–cortex system offer exciting possibilities for AI but also present significant challenges. Future brain-inspired AI systems could potentially have these capabilities:

1. Learning continuously without forgetting, adapting quickly to new situations while maintaining a stable knowledge base [5].
2. Handling noisy or incomplete data more effectively through pattern separation and completion mechanisms [9].
3. Engaging in episodic memory and mental time travel, enabling more context-rich decision-making and creativity [27].
4. Integrating emotions and value judgments for a more nuanced understanding of human preferences [55].

However, significant challenges remain:

1. Bridging the gap between biological and artificial neural networks [56].
2. Scaling brain-inspired principles to handle vast amounts of data and complex tasks.
3. Achieving the energy efficiency of the human brain in artificial systems [57].
4. Translating the temporal dynamics of biological neural networks into artificial systems [58].
5. Integrating various brain-inspired mechanisms into a cohesive system [59].
6. Determining the appropriate level of biological mimicry [8].
7. Addressing the ethical implications of advanced AI systems [60].

Despite these challenges, the potential benefits of brain-inspired AI are immense, promising to revolutionize fields from healthcare to environmental protection. Progress will likely come from interdisciplinary approaches, combining neuroscience insights with innovations in computer science and engineering. This journey toward more brain-like AI not only aims to create more powerful machines but also deepens our understanding of intelligence itself.

## 7. Testable Predictions

Table 1 presents a summary of key concepts in hippocampus-inspired AI along with their associated predictions and testable hypotheses. These concepts emerge from our analysis of hippocampal mechanisms and their potential AI analogues, offering a roadmap for future research in brain-inspired artificial intelligence. The table encapsulates seven primary areas where biological insights could drive innovation in AI systems, potentially leading to more adaptive and efficient learning algorithms.

These testable predictions provide concrete directions for empirical investigation, bridging the gap between theoretical neuroscience insights and practical AI advancements. By validating or refuting these hypotheses, researchers can not only drive progress in AI but also gain valuable insights into the computational principles underlying hippocampal function. This bidirectional flow of knowledge between neuroscience and AI has the potential to accelerate progress in both fields, bringing us closer to artificial systems that can learn and adapt with the flexibility and efficiency of biological intelligence.

## 8. Conclusions: Charting the Course for Brain-Inspired AI

The intersection of neuroscience and artificial intelligence offers unprecedented opportunities for advancing both fields. Through our exploration of the hippocampus–cortex system, we have identified several promising directions for AI development that could address fundamental challenges like the stability–plasticity dilemma. The complementary learning systems theory [3,5] provides a framework for balancing rapid learning with stable knowledge retention, potentially enabling AI systems that can learn continuously while maintaining accumulated knowledge.

Key innovations in AI inspired by hippocampal function could include the following:

- Dual-process learning systems that mirror hippocampal-cortical interactions [61]
- Memory consolidation mechanisms incorporating replay and offline learning [62]
- Pattern separation and completion capabilities for the robust handling of noisy or incomplete data [20,21]
- Hierarchical memory systems that support both episodic and semantic learning [39,40]
- Theta-coordinated learning mechanisms for error-driven adaptation [63]

Recent advances in AI, such as deep reinforcement learning [62] and large language models [64], demonstrate the potential for brain-inspired approaches. However, significant challenges remain in bridging the gap between biological and artificial neural networks [56]. These include achieving the energy efficiency of biological systems [57], implementing temporal dynamics [58], and addressing ethical considerations [60].

The path forward requires interdisciplinary collaboration between neuroscientists, computer scientists, and cognitive psychologists. As Hassabis and colleagues [27] argue, this bidirectional exchange between neuroscience and AI can deepen our understanding of intelligence while advancing technological capabilities. By drawing inspiration from the remarkable adaptability and efficiency of the hippocampus–cortex system, we may develop AI systems that better serve human needs while providing insights into our own cognitive processes.

## Figures and Tables

**Table 1:** Hippocampus-Inspired AI Concepts: Predictions and Testable Hypotheses.

Concept	Prediction	Testable Hypothesis
1. Dual-Process Learning	Outperform single-process systems in rapid adaptation and retention	Higher performance in continued learning with novel tasks
2. SWR–BARR Consolidation	More stable and generalizable learning	Decreased learning interference while improving skill adaptation
3. Context-Dependent Representations	Improved fine discrimination	Higher accuracy in visual recognition of similar stimuli
4. Time-Dependent Plasticity	More robust fine discrimination	Better retention in sequential learning tasks
5. Hierarchal Knowledge	Improved generalization and reasoning	Faster learning and better skill transfer in multi-task environments
6. Pattern Separation/Completion	Enhanced specific recall and generalization	Higher accuracy in few-shot transfer in multi-task environments
7. Oscillation-Inspired Training	More efficient learning and consolidation	Comparable performance with fewer iterations and improved stability
